# Early Fluvoxamine Reduces the Risk for Clinical Deterioration in Symptomatic Outpatients with COVID-19: A Real-World, Retrospective, before–after Analysis

**DOI:** 10.3390/microorganisms11082073

**Published:** 2023-08-12

**Authors:** Aristotelis Tsiakalos, Panayiotis D. Ziakas, Eleni Polyzou, Georgios Schinas, Karolina Akinosoglou

**Affiliations:** 1 Leto General, Maternity & Gynecology Clinic, 11524 Athens, Greece; 2 Department of Medicine, Brown University, Providence, RI 02912, USA; pd.ziakas@gmail.com; 3 Dept of Internal Medicine and Infectious Diseases, Medical School, University General Hospital of Patras, University of Patras, 26504 Rio, Greece; polyzou.el@gmail.com (E.P.); georg.schinas@gmail.com (G.S.); akin@upatras.gr (K.A.)

**Keywords:** coronavirus, COVID-19, SARS-CoV-2, fluvoxamine, vaccine

## Abstract

Fluvoxamine, a selective serotonin reuptake inhibitor with anti-inflammatory properties, has gained attention as a repurposed drug to treat COVID-19. We aimed to explore the potential benefit of fluvoxamine on outpatients with early SARS-CoV-2 infection. We performed a retrospective study of fluvoxamine adult outpatients with symptomatic COVID-19 disease of early onset (<5 days), in the context of an infectious diseases private practice, between September–December 2021, in Greece. Patients with disease duration ≥5 days, dyspnea and/or hypoxemia with oxygen saturation <94% in room air and pregnancy were excluded from the analysis. In total, 103 patients, 54 males/49 females with a median age of 47 years (39–56), were included in this study. Patient characteristics were balanced before and after the introduction of fluvoxamine. Two patients in the fluvoxamine arm (3.8%; 95% CI 0.4–13) had clinical deterioration compared to 8 patients in the standard of care group (16%; 95% CI 7.2–29.1, *p* < 0.04). After controlling for age, sex, body mass index > 30 and vaccination status, fluvoxamine was independently associated with a lower risk of clinical deterioration (adj. OR 0.12; 95% CI 0.02–0.70, *p* < 0.02). Adding on fluvoxamine to treatment for early symptomatic COVID-19 patients may protect them from clinical deterioration and hospitalization, and it is an appealing low-cost, low-toxicity option in the community setting and warrants further investigation.

## 1. Introduction

In March 2020, the World Health Organization declared the COVID-19 pandemic, caused by the SARS-CoV-2 virus, a global emergency. Since then, the number of persons affected worldwide has markedly increased, putting pressure on health systems and healthcare budgets worldwide. The clinical manifestations show a biphasic course of initial viral replication and toxicity, later followed by a phase of hyperinflammatory response, reflected in severe respiratory distress and commonly multiorgan failure [1]. In this context, early antiviral and later concomitant anti-inflammatory intervention represent the mainstay of therapeutic management in these patients. Much attention has been drawn to populations at risk for poor outcomes, including patients with multiple comorbidities, immunocompromised, over-aged, obese patients, and pregnant women who are at risk of progression to severe disease [2]. The presence of underlying systemic disease, on top of increased age, seems to have an additive effect on worsening outcomes [2]. Thus, early identification and prompt intervention in this context are pivotal to avoid rapid clinical deterioration and ensure optimal outcomes.

Currently, clinical research and experience have provided outpatients at risk for worse outcomes with successful therapeutic options embedded in living guidelines [3]. Current options include nirmatrevir/ritonavir, molnupiravir, or a 3-day course of remdesivir in the form of outpatient parenteral antimicrobial therapy [3]. However, national policies as to eligibility criteria pertaining to drug disposal and administration remain diverse, vastly due to associated cost, while global socio-economic inequalities do now ensure for worldwide distribution [4]. Divergence in medical technology in the absence of time for generic development calls for a parallel road of action in low and middle-income countries where stringent budgets drive health policies [5,6]. The idea of repurposing existing drugs is quite appealing in the setting of the COVID-19 pandemic, especially in limited-resource settings. Success has been variable, despite the effort and time spent by researchers in numerous clinical trials across more than 100 countries [7].

A number of prior observational cohort studies of patients with COVID-19 have reported a reduced number of deaths or need for mechanical ventilation in the acute care setting, with subsequent reduced risk of emergency department or hospital visits among those taking antidepressants [8]. Preclinical evidence has also demonstrated the in vitro efficacy of several SSRIs against various variants of SARS-CoV-2, both in human and nonhuman host cells [9]. Fluvoxamine, a selective serotonin reuptake inhibitor approved for the treatment of obsessive-compulsive disorder, is shown to have significant anti-inflammatory properties in animal models and in vitro studies [10,11,12]. Recent work supported the antiviral and anti-inflammatory properties of fluoxetine in a mouse model of SARS-CoV-2 infection and its in vitro antiviral activity against different variants of concern, including Omicron BA.5 [13]. The lung presents a high serotonin transporter expression in humans, suggesting that potent vasoconstrictor serotonin bioavailability is possibly primarily regulated by the serotonin transporter in the lung endothelium [14]. Thus, one can assume that antidepressants such as fluvoxamine may affect COVID-19 lung function via various mechanisms. Specifically, even before the COVID-19 pandemic, fluvoxamine has been shown to possess anti-inflammatory properties in murine models of septic shock [15]. This process is possibly mediated by the sigma-1 receptor pathway of cytokine release [12]. Another mechanism via which fluvoxamine exerts beneficial effects includes the acid sphingomyelinase/ceramide (ASM) pathway [12]. Inhibition of the ASM/ceramide system by specific antidepressants such as fluvoxamine or fluoxetine prevents infection of Vero E6 cells with SARS-CoV-2 [16]. When reconstitution of ceramides in cells is treated with these specific antidepressants, infection restoration occurs [16]. Other pathways include increased melatonin, modulation of lysosomal viral trafficking, platelet regulation, and activity against the endothelium [12].

Several randomized trials on outpatient COVID-19 cases implied a benefit in averting clinical deterioration and/or hospitalizations [17,18]. To date, however, the COVID-19 Treatment Guidelines Panel does not recommend either for or against its use, given the lack of sufficient evidence [3]. Nonetheless, the drug remains a compelling option for early COVID-19 due to its low cost, the oral route of administration, and the known safety profile, which may render fluvoxamine a cost-effective alternative to oral antivirals for COVID-19 [19,20]. Our study aims to investigate the effect of early fluvoxamine treatment in symptomatic outpatients with COVID-19, with a hypothesis that prompt fluvoxamine administration, as a supplementary treatment to the standard care, reduces the risk for clinical deterioration in these patients.

## 2. Materials and Methods

We performed a retrospective, before–after analysis of COVID-19 patient data, treated in an outpatient setting between September 2021 and December 2021, at a private infectious disease clinic in Athens, Greece. The analysis included adult patients living in the community with a confirmed SARS-CoV-2 polymerase chain reaction (PCR) or rapid antigen test who had a symptomatic infection with recent onset (<5 days). Inclusion criteria were the presence of fever >38 °C on at least one occasion and/or respiratory symptoms (cough and/or shortness of breath). Patients with long-lasting symptoms (≥5 days), existing comorbidities with impaired performance status before testing positive for SARS-CoV-2 or oxygen saturation of less than 94% at baseline assessment, as well as pregnant individuals, were excluded from this study since they were referred to allocated COVID-19 hospital services in the Athens metropolitan area. Patients were instructed to self-isolate, equip with a thermometer, a pulse oximeter, and a blood pressure monitor via a healthy relative or a proxy pharmacist, per the guidelines advice for mild and moderate disease [21]. During the first contact, eligible patients were instructed to start their treatment the same day. Before the introduction of fluvoxamine, patients were assigned to oral inhalation of budesonide 800 milligrams(mg) twice daily for 14 days and over-the-counter acetaminophen up to 4 times daily—maximum 4 grams (g) per day. Inhaled budesonide was shown to shorten the time to recovery, reduce emergency visits, have a potential benefit on hospitalizations and minimal side-effects [22,23,24], and it is a therapeutic option for early outpatient treatment of adult patients with COVID-19 per the National Public Health Organization algorithm [21]. Anticoagulation prophylaxis with low-molecular-weight heparin (enoxaparin 4000 IU SC once daily) was added for symptomatic high-risk patients with a history of thrombosis, known thrombophilia or cancer, obesity or advanced age (>65 years) in accordance with the local hematology association guidelines. Fluvoxamine was added as an off-label option after November 2021 at a dose of 100 mg twice daily for a 10-day course based on recently published evidence from two randomized-controlled trials [17,18]. Patients were instructed to provide daily updates of their status on a 24/7 basis. On day 5 after initiation of symptoms, treated patients were instructed to have a laboratory assessment including laboratory inflammation markers, cardiac enzymes and thrombosis biomarkers: a complete blood count, C-reactive protein (CRP) and ferritin levels, troponin, creatine phosphokinase (CPK) and lactate dehydrogenase levels and a quantitative D-dimer assay [25,26,27,28]. All patients were screened by phone and/or e-mail and provided informed consent, typically through electronic means.

The primary outcome was clinical deterioration defined by worsening dyspnea with oxygen saturation <94% on room air and the development of pneumonia with or without the need for hospitalization. Data on patients referred to hospital care were updated after discharge.

We analyzed data using Stata 17 (College Station, TX, USA) software. Continuous variables were compared using the non-parametric Mann–Whitney test. For categorical outcomes, Fisher’s exact test was used. To measure the association of fluvoxamine with outcome, we reported Odds Ratios (OR) with 95% confidence intervals (95% CIs) using a modified logistic regression framework to compensate for small samples and rare events [29]. Significance was set to α< 0.05.

## 3. Results

Between 1 September 2021 and 31 December 2021, a total of 129 adults with a positive PCR or rapid antigen test for COVID-19 were evaluated. A total of 20 were excluded upon initial screening (9 asymptomatic and 11 pregnancies); six denied receiving the proposed treatment (2 in the standard of care and 4 in the fluvoxamine arm). Of 129 screened, 103 adults (54 male, 52%) were finally analyzed. Their median age was 47 years (range 39–56), with 15% over 65. Fifty (49%) had an underlying chronic condition, including obesity (22% with BMI > 30), hypertension (17%), diabetes mellitus (11%), autoimmunity (8%), dyslipidemia (9%), neurologic disorder (5%) or cancer (4%). Seventy-eight (76%) were fully vaccinated, and 26 (25%) had received a booster shot.

A total of 53 patients initiated fluvoxamine treatment at a dose of 100 mg bid. There were no statistically significant differences in patient demographics, presentation, standard of care and comorbidities after the introduction of fluvoxamine, as shown in Table 1, except for obesity, which was more prevalent in the fluvoxamine group (28% vs. 14%, with marginal significance *p* = 0.10).

All patients were followed from COVID-19 documentation to the resolution of symptoms or deterioration requiring hospital care. Eight patients (7%) were hospitalized, six due to progressive hypoxemia and one due to hypoxemia and acute deep vein thrombosis. One required mechanical ventilation and ICU support. All 8 patients were discharged from the hospital and were alive on the last follow-up. Two additional patients developed hypoxemia and pneumonia and were treated successfully as outpatients with empirical antimicrobial coverage. Of the 10 patients with the primary outcome, two patients (3.8 percent; 95% CI 0.4 to 13.0) had a clinical deterioration in the fluvoxamine group vs. eight (16 percent; 95% CI 7.2 to 29.1) in the standard of care, corresponding to a lower risk for clinical deterioration (crude Odds Ratio 0.24; 95% confidence interval 0.06–1.05, *p* 0.06). For a baseline risk of 16% for clinical deterioration in the standard care group, the calculated number needed to treat to benefit (NNTB) was 9, with a wide 95% confidence interval (4.2 to 118.6). After age–sex adjustment, BMI and vaccination status controlling (Table 2), the use of fluvoxamine showed significant protection against clinical deterioration (adjusted Odds Ratio 0.12; 95% confidence interval 0.02–0.70, *p* 0.02). Vaccination was protective, and obesity adversely correlated with outcome.

Laboratory assessment on day 5 showed no significant differences between treatment arms in complete blood counts and biochemistry parameters, except for lymphocyte count (Table 3). 

In the fluvoxamine group, 50/53 (94%) completed 10 days of therapy. Seven patients (13%) reported dizziness and nausea during fluvoxamine treatment, which led to early discontinuation (<5 days) in four patients (8%). 

## 4. Discussion

In this retrospective before–after analysis, we found that add-on fluvoxamine to the standard of care was associated with a reduction in clinical deterioration for symptomatic outpatients with confirmed COVID-19. Four percent of patients taking fluvoxamine had a clinical deterioration as opposed to 16 percent of patients who did not. This study, albeit small and retrospective, adds to the recent literature that implies a benefit of using fluvoxamine to treat COVID-19 in the outpatient setting. While our findings align with previous research, our study contributes to the existing literature by providing real-world evidence, which is crucial for validating findings from controlled settings.

A number of previous studies have provided supportive evidence that oral fluvoxamine cuts the risk for clinical deterioration when used early after COVID-19 diagnosis in the outpatient setting with an excellent safety profile and limited side effects, including mild nausea, abdominal discomfort and sleep disturbances [17,18]. In a placebo-controlled study (STOP COVID trial) in the St Louis metropolitan area, none out of 80 patients randomized to fluvoxamine (100 mg up to 3 times daily for 15 days) showed clinical deterioration compared to 6 of 72 (8.3%) patients in the placebo group [17]. Even though statistical differences were detected, the sample size was small; hence, conclusions were rather fragile. A second larger randomized-controlled study of oral fluvoxamine (100 mg twice daily for ten days) across 11 clinical sites in Brazil (TOGETHER trial) found that the composite outcome of either requiring observation in an emergency setting or admission to a tertiary hospital was lower in the fluvoxamine arm (79/741, 11%) compared to the placebo arm (119/756, 16%) [18]. In this setting, prolonged observation was used as a proxy for hospitalization because hospital beds were fully occupied during this phase of the pandemic in Brazil. However, when viral clearance, all-cause hospitalization, time to hospitalization, number of days in the hospital, time to recovery, days of mechanical ventilation or mortality were examined, no significant differences between fluvoxamine and placebo were observed [18,30]. In comparison to these findings, a follow-up study from the same group investigating the combination of fluvoxamine and inhaled budesonide observed a significant reduction in the composite outcome of emergency setting retention for COVID-19 or hospitalization due to disease progression compared to placebo (1.8% vs. 3.7%; relative risk 0.50, 95% credible interval 0.25 to 0.92) [31]. A third, retrospective cohort US study of deidentified electronic health record data across 87 health centers used propensity score matching and found that fluoxetine or fluvoxamine may reduce mortality from COVID-19 from 13.3% in matched controls to 10% in the fluoxetine or fluvoxamine treated (a significant relative risk reduction by 26%) [8]. This finding came in agreement with other authors from smaller studies finding reduced mortality in patients receiving fluvoxamine, especially the earlier given in the course of the disease [32,33,34,35]. Recent systematic reviews and meta-analyses confirmed that fluvoxamine significantly and substantially reduces hospitalization risk among outpatients with COVID-19 [35,36,37,38]. Benefits also extended to patients admitted to intensive care units. A prospective cohort study of critically ill COVID-19 patients reported a significant association between the 15-day use of fluvoxamine prescribed at a daily dose of 300 mg and reduced mortality [39]. Real-world data from Africa, including 316 patients, of whom 94 received fluvoxamine in addition to standard care, showed that fluvoxamine use was significantly associated with reduced mortality (AHR = 0.32; *p* < 0.001, NNT = 4.4) and with increased complete symptom resolution (AOR = 2.56; *p* < 0.001, NNT = 4.44) [15]. These effects were irrespective of clinical characteristics, including vaccination status. There was a trend toward greater side effects with fluvoxamine (7.45% vs. 3.15%; *p* = 0.06); however, the majority were mild in severity, and none required regimen discontinuation. A ten-day course of 100 mg per day was well tolerated and related to reduced mortality and increased symptom resolution without an increase in time to hospital discharge [15]. Nonetheless, psychotropic drugs, including other selective serotonin reuptake inhibitors, often lack a significant or similar association with outcomes [8,33,40]. The reason for this discrepancy may lie in underlying mechanisms of action [12]. 

In turn, the COVID-OUT trial failed to show a significant effect of fluvoxamine in preventing disease progression with an adjusted odds ratio of 0.94 (95% CI, 0.66 to 1.36) for the composite outcome of SpO2 of 93% or less on room air, emergency department visit, hospitalization or death [41]. In their subsequent long-term follow-up report, the authors found that there was no significant effect on long COVID incidence with fluvoxamine compared to placebo after 10 months [42]. Likewise, a recent study by McCarthy et al. failed to show any benefits, reporting results from the Accelerating COVID-19 Therapeutic Interventions and Vaccines (ACTIV-6) platform randomized clinical trial [43]. Similar to our study, high-risk comorbidities, including obesity, hypertension, asthma and diabetes, were common, and 67% of patients had received at least two vaccine doses. Results showed no significant benefit as to the median time to sustained recovery, similar to comparable rates of hospitalization, urgent care visit, emergency department visit or death through day 28 [43]. 

Interpretation of these conflicting results should be made with caution. The reason for this variability in many studies lies in their diverse geographic locations, different circulating variants, time periods, variable baseline risks of progression to severe disease and vaccination rates. Some also used differing dosing regimens and assessed different clinical outcomes, and their utility strongly differs between doctors and patients. Using composite outcomes can enhance statistical methods but also prove problematic. Using patient-centered and reported outcomes such as disability, discomfort and death is more meaningful than outcomes of health resource utilization or their proxies, which can be influenced by institutional bed availability and individual caregiver practices. Another explanation could be owing to the change in the severity of COVID-19 over different dominating variants. Hence, currently, the less severe disease does not easily allow for clinical difference detection, even though we managed to do so.

Despite potential benefits, a note of caution should be added concerning potential drug–drug interactions when prescribing for fluvoxamine [44]. The latter is a potent modulator of cytochrome P450 family enzymes. Hence, its administration can variably increase exposure to co-medications metabolized by this enzyme [45]. Candidate population usually bears multiple comorbidities, and polypharmacy remains an issue. Although the duration of administration is short, CYP inhibition is expected to occur immediately. Hence, the potential need for titration of co-medication (e.g., antiepileptics, neuroleptics, etc.) based on expected clinical response should not be ignored, since dose adjustment may potentially destabilize the patient. Serotonin toxicity, as well as QTc prolongation, must also be screened for in these patients. Other adverse events, including gastrointestinal disturbances, are minor and rarely lead to regime discontinuation.

Considering our analysis in combination with safety data, worldwide accessibility and the current price of approximately USD 1 per day [46], fluvoxamine may be a reasonable option for high-risk outpatients who do not have access to SARS-CoV-2 direct antivirals, monoclonal antibodies or compassionate use in the context of clinical trials. It is estimated that even at a number to treat of 200 patients (i.e., ARR of 0.5%), the corresponding cost to prevent admission would only be USD 2800 [35]. In a recent cost–consequence model, administering a 10-day course of fluvoxamine to COVID-19 outpatients at high risk for poor outcomes is substantially cost-saving, saving USD 232 and 0.15 hospitalization days per patient compared with standard of care in the US [47]. However, as previously stated, need for hospitalization, ICU care and post-acute covid syndrome-related expenditure, which mainly drives costs in such models, can be misleading [47].

That said, our study also highlighted the importance of obesity and vaccination on COVID-19 outcomes. Obesity is a well-recognized risk factor for poor outcomes in patients with COVID-19 [48,49]. In a recent meta-analysis including a total of 208 studies with 3,550,997 participants from over 32 countries, patients with obesity were at increased risk of both COVID-19-related hospitalizations (OR 1.72) and death (OR 1.25). Extreme obesity increases the risk of COVID-19-related hospitalizations and death (OR 2.53 and 2.06, respectively) [49]. This can be attributed to several reasons. First, adipose tissue is rich in ACE2 receptors, which represent a port of entry for SARS-CoV-2 to human cells and a consequent reservoir for viral replication [50]. Second, obesity per se has been shown to cause immune dysregulation and increase the susceptibility to infection while increasing inflammation of the parenchyma and bronchi [51]. Last, obesity decreases lung capacity and reserve, making mechanical ventilation even more difficult. 

Vaccination has been found to have a protective effect in our cohort of patients with regard to progression to severe disease. This comes as no surprise as vaccination roll-out programs, including primary series and booster doses, have halted the course of the pandemic worldwide. Vaccine effectiveness for the primary series at baseline was 92% for hospitalizations and 91% for mortality, while for booster doses, vaccine effectiveness at baseline was 70% against infections and 89% against hospitalizations [52]. At the time this study was carried out, booster shots had just begun to be recommended and performed in Greece, and the expected vaccine efficacy from primary doses was 79% and 86% for hospitalizations and mortality, respectively. Even so, completion of the primary series seems to provide adequate protection against progression to severe disease and adverse outcomes.

Last, we also showed that patients receiving fluvoxamine presented a faster restoration of lymphocyte count levels. Severe lymphopenia is a well-recognized marker for the severity of COVID-19 disease [53,54]. It has been associated with a high prevalence of known risk factors for worse outcomes, as highlighted in many studies [55]. It has also been associated with a hyper-inflammatory response characterized by increased serum levels of acute phase proteins and pro-inflammatory mediators [56]. The exact reasons for such an event remain elusive. However, possible explanations could be that SARS-CoV-2 directly infects replication of inflammatory monocytes and lymphocytes, leading to apoptosis of T lymphocytes in vitro.

Additionally, SARS-CoV-2 has been detected in the peripheral blood mononuclear cells and postmortem lung tissues of COVID-19 patients [57]. There is increasing evidence of bone marrow suppression due to SARS-CoV-2 infection of hematopoietic stem cells [58], while thymus suppression has also been proposed [59]. Lymphopenia recovery is variable in COVID-19 patients. Previous studies have shown that lymphopenia may persist for weeks after acute disease [60]. Recently, published data have provided evidence of increased mortality in COVID-19 patients with persistently decreased levels of absolute lymphocyte count, while survivors experience lymphocyte recovery with sepsis [61]. Residual immunosuppression may be a cause of secondary infections and worse outcomes.

There are several limitations to be reported in this study. It is a retrospective cohort study with a pre-post analysis that cannot compete with a randomized trial recruiting patients prospectively. Additionally, the size of the study and the rarity of the primary outcome, especially in the fluvoxamine arm, render a multivariable analysis a challenge. To correct for bias related to small size and rare events, we used a penalized maximum likelihood estimation method to adjust for confounders [62,63], yet the effects are fragile, as seen with previous fluvoxamine studies [17]. Finally, a significant association between fluvoxamine and clinical deterioration may imply but does not prove a causal link, as complex clinical interactions may be present and spurious associations can arise. Despite its limitations, our study adds to the plausible evidence that fluvoxamine at least warrants further investigation in the treatment of COVID-19. Our analysis spans 4 months of observation in a private practice setting in the Athens metropolitan area, with a uniform handling of outpatients compliant with the local guidelines [21], resulting in well-balanced characteristics before and after fluvoxamine was added. Furthermore, the study is an example of the shift towards a more pragmatic approach of minimal in-person contact and self-reported outcomes, a growing and efficient practice during the pandemic [17,64,65]. 

## 5. Conclusions

The combination of the low-cost, oral route of administration with limited adverse events renders fluvoxamine a promising candidate for early outpatient use, particularly in limited resource settings. Furthermore, the anti-inflammatory properties of fluvoxamine are not expected to be related to circulating COVID-19 variants and may be useful regardless of the dominant strain. The track record of repurposing drugs to combat COVID-19 was of limited success, but fluvoxamine will remain in the spotlight insofar as supporting evidence accumulates. Our study contributes to the existing body of literature by offering robust real-world evidence that underscores the efficacy of early administration of fluvoxamine as an adjuvant treatment for COVID-19 and suggests a potential immunomodulatory role for its use in this context.

## Figures and Tables

**Table 1:** Patient characteristics and outcome before and after initiating fluvoxamine as an add-on to the standard-of-care (SOC), early outpatient care for symptomatic COVID-19.

	Fluvoxamine + SOC (N = 53)	SOC (N = 50)	*p*
Median Age (years)	45 (IQR 41–55)	49 (IQR 39–60)	0.65
Male Gender n (%)	25 (47)	29 (58)	0.33
BMI (kg/m^2^)			
Median	26.4 (24.0–31.3)	25.8 (23.6–28.3)	0.25
Comorbidities n (%)			
Obesity (BMI > 30)	15 (28)	7 (14)	0.10
Hypertension	10(19)	7 (14)	0.60
Diabetes	5(9)	6(12)	0.76
Dyslipidemia	6(11)	3(6)	0.49
Autoimmunity	5(9)	3(6)	0.72
Neurologic	3(9)	2(4)	1
Thrombophilia	3(6)	0	0.24
Cancer	3(6)	0	0.24
COPD	1(2)	1(2)	1
Other	28(53)	22(44)	0.43
Vaccination n (%)			
Fully vaccinated	38 (72)	40 (80)	0.37
Booster shot	16 (30)	10 (20)	0.26
Treatment n (%)			
Enoxaparin prophylaxis	15 (28)	11 (22)	0.50
Budesonide	53 (100)	50 (100)	1
Outcome n (%)			
Clinical deterioration	2 (4%)	8 (16%)	0.048
Hospitalization/ICU	1/0	6/1	0.05
Deaths	0 (0)	0 (0)	-

BMI: Body Mass Index; ICU: Intensive Care Unit; IQR: Interquartile Range.

**Table 2:** Association of fluvoxamine use with clinical deterioration, crude (unadjusted) and adjusted estimates.

All Patients (n = 103)	Odds Ratio	95% Confidence Interval	*p*
Unadjusted effect			
Fluvoxamine	0.24	0.06–1.05	0.06
Adjusted effects			
Fluvoxamine	0.12	0.02–0.70	0.02
Age	1.05	0.99–1.11	0.08
female gender	0.82	0.20–3.34	0.77
BMI > 30	8.2	1.55–43.11	0.01
Full vaccination	0.17	0.03–0.84	0.03

Odds Ratio < 1 denotes a favorable effect.

**Table 3:** Comparison of laboratory profile on day 5.

	Fluvoxamine + SOC (N = 53)	SOC (N = 50)	*p*
Complete blood count (CBC)			
White Blood Cell count (/μL)	5520 (IQR 4680–6300)	5383 (IQR 4500–6600)	0.75
Lymphocyte count (/μL)	1862 (IQR 1451–2211)	1638 (IQR 1210–2005)	0.03
Lymphocyte count < 1000 (/μL) ^†^	1(2%)	4(8%)	0.20
Hemoglobin (g/dL)	14 (IQR 13.1–15.0)	14 (IQR 12.6–15.2)	0.49
Platelet count (×10^3^/μL)	214 (IQR 180–267)	199 (171–276)	0.63
Biochemistry/Biomarkers			
C-reactive protein (mg/L)	4 (IQR 2.4–8.9)	4.6 (IQR 3.1–16)	0.15
C-reactive protein > 5 mg/L	22 (42%)	23 (46%)	0.69
C-reactive protein > 100 mg/L ^†^	0	3 (20%)	0.11
Serum ferritin (ng/mL)	132(IQR 72–199)	122(IQR 70–188)	0.9
Serum ferritin > 1000 (ng/mL) ^†^	0	1(2%)	0.49
Lactate dehydrogenase (LDH) U/L	165(IQR 140–201)	166 (IQR 118–193)	0.43
Creatine phosphokinase (CPK) U/L	87(66–115)	77(44–118)	0.20
Troponin assay > 0.40 ng/mL	0	0	
D-dimer assay > 500 μg/mL	9 (17%)	16 (32%)	0.11

^†^ Cut-off designated as laboratory evidence of severe COVID-19 disease by local guidelines [21].

## Data Availability

The data presented in this study are available on request from the corresponding author. The data are not publicly available due to privacy and ethical constraints.
